# In Truth “Docile Ruminants in a Herd”

**Published:** 1910-11

**Authors:** Dan Millikin

**Affiliations:** Hamilton, Ohio


### In Truth "Docile Ruminants in a Herd."

HAMILTON, OHIO, October 5, 1910.

*Editor Lancet-Clinic:*

Your correspondent, Dr. Kramer, is usually up to date, but his communication of September 27 shows that for once he has not heard the news. He declares that he "has no desire to see the State society destroyed," when, as a matter of fact, that organization committed suicide some years ago, and was buried in a shallow grave by its sole heir and administrator, the Ohio State Medical Association.

The difference between the old society and the present association lies chiefly in the fact that the former was a natural and inevitable coming together of scientific gentlemen in response to mutual attraction, whereas the association is an aggregation induced by extraneous and artificial forces. That was a beautiful growth; this is a machine.

The present artificial organization, designed and built to drive all the physicians of the State in a corral, is an ingenious thing, and admirable while it works; but when the physicians of the State happen to observe that they are held to be docile ruminants in a herd, they will stampede, and after the dust has blown away there will be a reorganization—possibly a resurrection. I hope to meet you on the grounds with Dr. Kramer.

DAN MILLIKIN.

Same here in Texas.—*Daniel.*

